# Development of Physiologically Responsive Human iPSC-Derived Intestinal Epithelium to Study Barrier Dysfunction in IBD

**DOI:** 10.3390/ijms21041438

**Published:** 2020-02-20

**Authors:** John P. Gleeson, Hannah Q. Estrada, Michifumi Yamashita, Clive N. Svendsen, Stephan R. Targan, Robert J. Barrett

**Affiliations:** 1 Board of Governors Regenerative Medicine Institute, Cedars-Sinai Medical Center, Los Angeles, CA 90048, USA; jgleeson@cmu.edu (J.P.G.); Hannah.Estrada@cshs.org (H.Q.E.); Clive.Svendsen@cshs.org (C.N.S.); 2 Department of Pathology and Laboratory Medicine, Cedars-Sinai Medical Center, Los Angeles, CA 90048, USA; Michifumi.Yamashita@csmc.edu; 3 Department of Biomedical Sciences, Cedars-Sinai Medical Center, Los Angeles, CA 90048, USA; 4 F. Widjaja Foundation Inflammatory Bowel and Immunobiology Research Institute, Cedars-Sinai Medical Center, Los Angeles, CA 90048, USA; Stephan.Targan@cshs.org

**Keywords:** intestinal permeability, human intestinal organoids, barrier function, disease modeling

## Abstract

In inflammatory bowel disease (IBD), the intestinal epithelium is characterized by increased permeability both in active disease and remission states. The genetic underpinnings of this increased intestinal permeability are largely unstudied, in part due to a lack of appropriate modelling systems. Our aim is to develop an *in vitro* model of intestinal permeability using induced pluripotent stem cell (iPSC)-derived human intestinal organoids (HIOs) and human colonic organoids (HCOs) to study barrier dysfunction. iPSCs were generated from healthy controls, adult onset IBD, and very early onset IBD (VEO-IBD) patients and differentiated into HIOs and HCOs. EpCAM+ selected cells were seeded onto Transwell inserts and barrier integrity studies were carried out in the presence or absence of pro-inflammatory cytokines TNFα and IFNγ. Quantitative real-time PCR (qRT-PCR), transmission electron microscopy (TEM), and immunofluorescence were used to determine altered tight and adherens junction protein expression or localization. Differentiation to HCO indicated an increased gene expression of *CDX2*, *CD147,* and *CA2*, and increased basal transepithelial electrical resistance compared to HIO. Permeability studies were carried out in HIO- and HCO-derived epithelium, and permeability of FD4 was significantly increased when exposed to TNFα and IFNγ. TEM and immunofluorescence imaging indicated a mislocalization of E-cadherin and ZO-1 in TNFα and IFNγ challenged organoids with a corresponding decrease in mRNA expression. Comparisons between HIO- and HCO-epithelium show a difference in gene expression, electrophysiology, and morphology: both are responsive to TNFα and IFNγ stimulation resulting in enhanced permeability, and changes in tight and adherens junction architecture. This data indicate that iPSC-derived HIOs and HCOs constitute an appropriate physiologically responsive model to study barrier dysfunction and the role of the epithelium in IBD and VEO-IBD.

## 1. Introduction

Inflammatory bowel disease (IBD) refers to a spectrum of complex polygenic disorders that are thought to result from dysregulated immune responses to commensal microbes in genetically susceptible hosts. In IBD, the intestinal epithelium is characterized by increased permeability both in active disease and remission states, and this increased permeability has been associated with elevated risk of relapse [1,2,3]. Increased permeability may lead to the translocation of luminal microbes to the host thereby initiating and propagating the dysregulated response in IBD. While it is known that various inflammatory cytokines and microbes can increase permeability, the finding of increased permeability among a proportion of unaffected first-degree relatives of IBD patients suggests a genetic association [4,5,6]. However, determining whether the cause of this increased permeability is due to a genetic component or inflammatory cytokines/microbes is challenging due to the complex microenvironment *in vivo*. Given there is increased permeability in both the small [1] and large intestine [3], coupled with enormous genetic heterogeneity among IBD patients, there is an urgent need to develop personalized *in vitro* cellular models to allow each of these components to be studied in isolation.

Previous studies have used human intestinal organoids (HIOs) to model intestinal permeability [7,8]. HIOs are complex three dimensional structures that contain all the intestinal epithelial subtypes and can be generated from either induced pluripotent stem cell (iPSCs) [9,10] or intestinal biopsies [11,12]. An advantage of using organoids is that they can be cultured for prolonged periods under tightly controlled conditions thereby allowing an examination of permeability under basal conditions and a subsequent analysis of permeability in response to various stimuli. A disadvantage of organoid culture is that they are heterogeneous both in terms of shape and size which may introduce variability to studies. Furthermore, they are polarized towards the lumen, meaning fluorescent dyes such as FITC-dextran must be microinjected inside them, which is technically challenging. Finally, while iPSC-derived HIOs, which have been shown to be representative of the small bowel [13] have been used in permeability studies, no studies have examined if iPSC-derived human colonic organoids (HCOs) are suitable for permeability studies.

To develop this personalized intestinal permeability model, we utilized HIOs derived from iPSCs. This cellular source of organoids was chosen as iPSCs can be generated from almost any individual [14] and previous reports demonstrate that iPSCs can be differentiated into both small [13] and large intestinal organoids [15,16]. In addition, there are repositories of genotyped lymphoblastoid cell lines (LCLs) generated from IBD patients. Given we have previously shown that we can reprogram LCLs to iPSCs [10], it would allow us to utilize these biorepositories to allow an examination of how genetic variations associated with IBD [17] would intrinsically affect intestinal permeability and subsequently influence their response to various cytokines. To confirm the feasibility of our approach and its applicability to the IBD field, we generated iPSCs from control individuals, adult onset IBD patients, and very early onset IBD (VEO-IBD) patients. We developed a protocol to incorporate epithelial cells derived from HIOs onto Transwells to standardize our approach and to show that permeability can be measured in all cell lines either under basal conditions or in response to inflammatory cytokines. Given that the large intestine is primarily affected in VEO-IBD patients [18,19], we directed iPSCs from these individuals to form HCOs and demonstrate their colonic phenotype. We subsequently show permeability can be measured in these colonic cells, ultimately demonstrating the feasibility of our approach whereby epithelial cells from HIOs and HCOs, derived from patient specific iPSCs, can be used to measure permeability under basal and inflammatory conditions.

## 2. Results

### 2.1. HIO-Derived Epithelium Becomes More Permeable When Exposed to Pro-Inflammatory Cytokines

**Figure 1:**
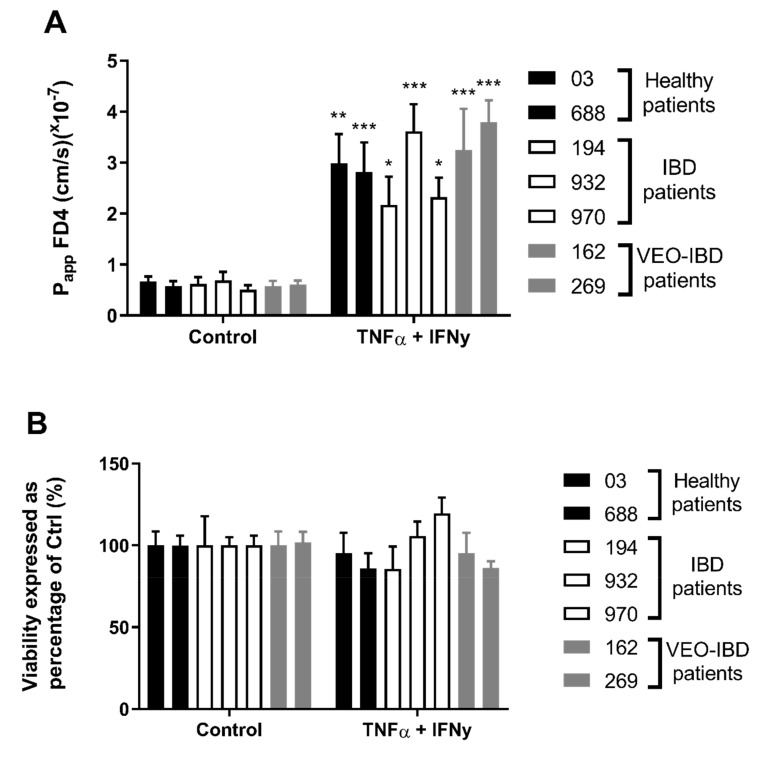
Effect of basolateral side addition of both TNFα and IFNγ (10 ng/mL) for 72 h on **A**) P_app_ of apically-added FITC-Dextran 4kDa (FD4) across HIO-derived epithelium obtained over 120 min. **B**) MTS viability indicated no cytotoxic effect from exposure to TNFα and IFNγ. Two-way ANOVA with Bonferroni’s multiple comparisons; * *p* < 0.05, ** *p* < 0.01, *** *p* < 0.001 compared with respective control. Each value represents the mean ± SEM, *n* = 3, with 3 independent replicates.

iPSCs from healthy controls (03i and 688i), adult-onset IBD (194i, 932i and 970i), and VEO-IBD (162i and 269i) patients were directed to form HIOs and were cultured for 20–30 days in a three-dimensional matrix. Given that iPSC-derived HIOs contain both an epithelial and mesenchymal cell population and we wished to seed only epithelial cells onto Transwells, HIOs were subsequently disassociated to a single cell suspension and epithelial cell adhesion molecule (EpCAM/CD326) was used to positively select for HIO-derived epithelial cells using magnetic-activated cell sorted (MACS). 2 × 10^5^ EpCAM+ cells were incorporated into 0.33 cm^2^ Transwell inserts and after 18 days, monolayers had a TEER of greater than 250 Ω·cm^2^ with no significant differences between basal Transepithelial electrical resistance (TEER) across all lines. After incubation of FD4 for 120 min the apparent permeability (P_app_) was determined, and a one-way ANOVA indicated no significant difference between the basal P_app_ across the different groups ranging from 0.57–0.77 × 10^−7^ cm/s (Figure 1A). TEER was unaffected by the addition of TNFα and IFNγ for 72 h (data not shown), however, P_app_ of FD4 was significantly increased across all lines 1.94–3.80 × 10^−7^ cm/s (*p* < 0.05–0.001). Similarly, there was no statistical difference between groups P_app_ when treated with cytokines. Increased permeability due to cytokine exposure was not due to cytotoxicity as the MTS cell viability assay showed no significant difference between control and treatment groups (Figure 1B). This data showed epithelial cells from iPSC-derived HIOs, generated from control individuals and a range of IBD patients, can successfully be seeded onto Transwell inserts, that intrinsic permeability can be measured under control conditions and that changes in permeability in response to cytokines can be quantified.

### 2.2. HCO-Derived Epithelium Reflects a Colonic Phenotype in Both Gene Expression and TEER

**Figure 2:**
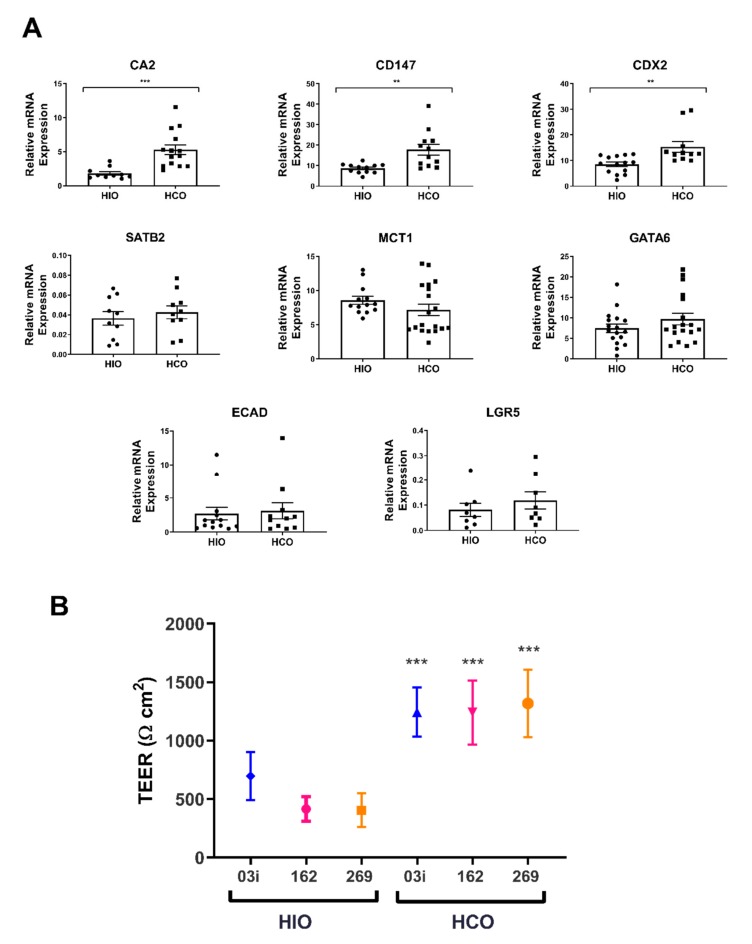
(**A**) qRT-PCR quantification of gene expression for colonic (*CD147*, *CDX2*, *MCT1*, *CA2*), and genes found throughout intestinal tract (*GATA6, ECAD* and *LGR5*). Relative gene expression is shown as percentage of GAPDH. Students unpaired *t* test, ** *p* < 0.01, *** *p* < 0.001, **** *p* < 0.0001 compared with HIO. Each value represents the mean ± SEM, *n* = 3 (03i, 162i, 269i), with 3 independent replicates of each cell line. (**B**) Basal transepithelial electrical resistance (TEER) of HIO- and HCO-derived epithelium grown on Transwell inserts for 18 days. Two-way ANOVA with Bonferroni’s multiple comparisons; * *p* < 0.05, ** *p* < 0.01, *** *p* < 0.001 compared with respective control. Each value represents the mean ± SD, *n* = 3, with 6 independent replicates.

Previous reports have shown that iPSCs can be directed to form HCOs by modifying the HIO differentiation protocol [15,16]. To confirm this finding, we generated both HIOs and HCOs from one control line and the two VEO-IBD lines, dissociated them to a single cell suspension, positively selected for EpCAM+ cells via MACS and cultured them either in a three dimensional matrix to generate epithelial only- HIOs (eHIOs) and HCOs (eHCOs) or as monolayers in Transwell inserts. eHIOs and eHCOs were cultured for 7 days and subsequently assessed for a panel of markers that were preferentially expressed in the large intestine. There was significantly increased expression of *CDX2* (*p* < 0.01) and the colonic markers *CD147* (*p* < 0.01) and *CA2* (*p* < 0.0001) in eHCOs as compared to HIOs although surprisingly, there was no significant difference in *MCT1* and *SATB2* (Figure 2A). We also examined for genes that are expressed throughout the intestinal tract, and as expected there was no difference in the expression of the zinc finger transcription factor *GATA6*, adherens junctional protein *ECAD,* and the intestinal stem cell marker *LGR5* (Figure 2A).

Furthermore, EpCAM+ cells from both HIOs and HCOs were seeded onto Transwell inserts and basal TEER was measured after 18 days. There was significantly higher TEER in monolayers derived from HCOs as compared to monolayers derived from their respective HIOs; 03i (1245 ± 33 vs. 615 ± 39 Ω·cm^2^), 162i (1242 ± 76 vs. 416 ± 17 Ω·cm^2^), and 269i (1319 ± 43 vs. 404 ± 16 Ω·cm^2^) (Figure 2B) (all *p* < 0.001) (Figure 2B). There is a 2–3-fold increase in TEER between HCO and HIO epithelium which reflects similar reported fold-increases between human and rodent tissues measured in Ussing chambers (Table 1). This data suggested that HCO epithelium has a gene expression profile reflective of colonic tissue and has a higher basal TEER, indicating a colonic physiology.

### 2.3. Pro-Inflammatory Cytokines Induce a Concentration-Dependent Increase in Permeability in both HIO and HCO Epithelium

**Figure 3:**
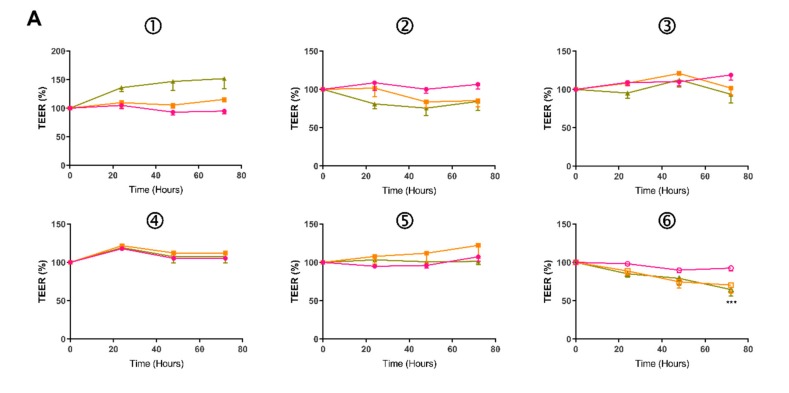

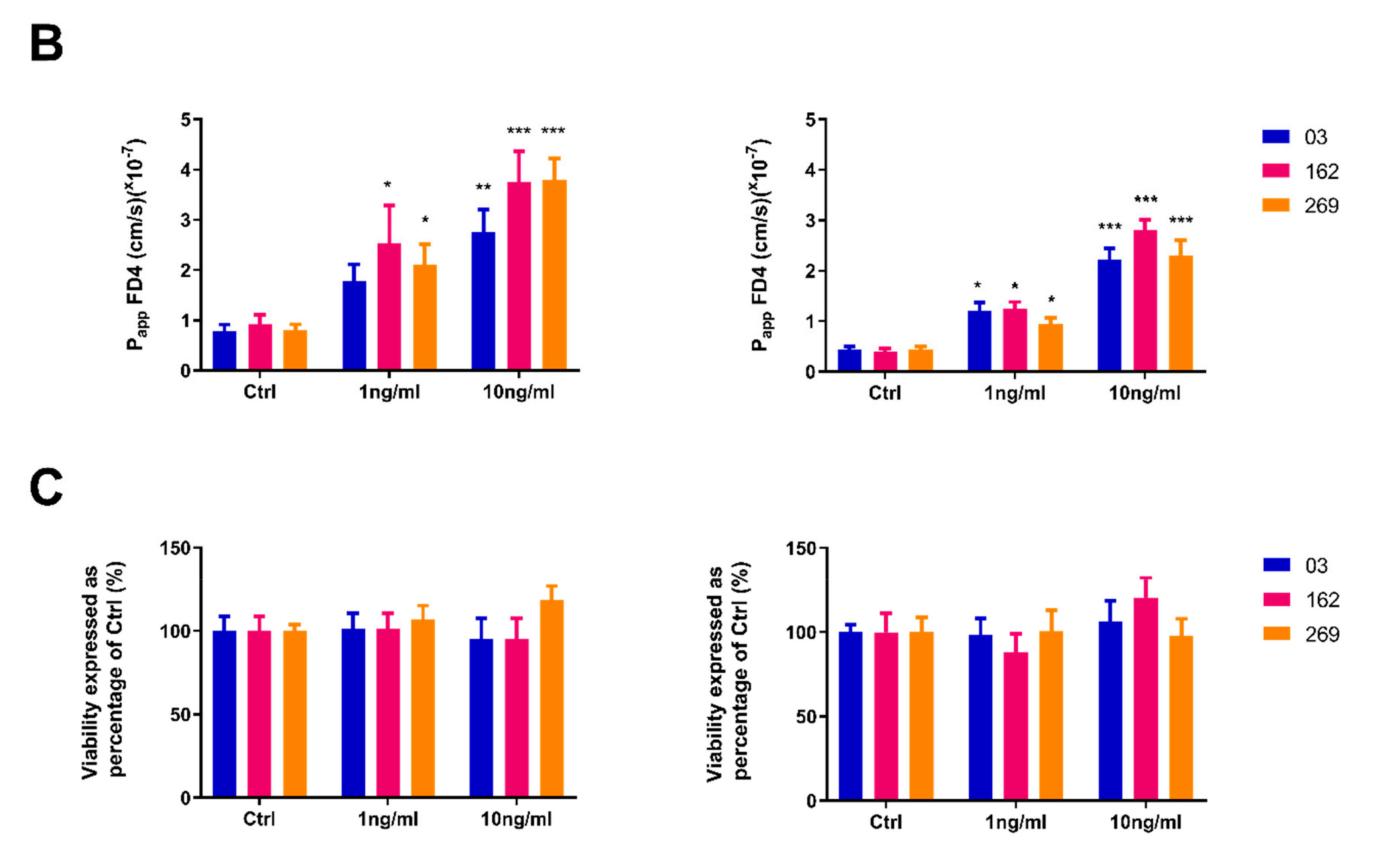
Effect of basolateral side addition of both TNFα and IFNγ (1 and 10 ng/mL) for 72 h on (**A**) % TEER values measured across HIO (1, 2, 3) and HCO (4, 5, 6) epithelium grown on Transwell inserts. (**B**) P_app_ of apically-added FD4 across HIO-/ and HCO-derived epithelium obtained over 120 min. **C**) MTS viability indicated no cytotoxic effect from exposure to TNFα and IFNγ. Two-way ANOVA with Bonferroni’s multiple comparisons; * *p* < 0.05, ** *p* < 0.01, *** *p* < 0.001 compared with respective control. Each value represents the mean ± SEM, *n* = 3, with 3 independent replicates.

To confirm the feasibility of utilizing iPSC-derived colonic epithelium for permeability studies, EpCAM+ positive cells from 03i, 162i and 269i lines were seeded onto Transwell inserts and cultured for 18 days. 0, 1, or 10 ng/mL of TNFα and IFNγ was then administered basally for 72 h. As shown in Figure 3, the basal TEER of HCO was higher, however, exposure to cytokines for 72 h had no major effect on % TEER of HIO or HCO (Figure 3A) with the exception of 269i HCO which had a reduction of 22% (Figure 3A6). Basal HIO P_app_ was 0.78–0.92 × 10^−7^ cm/s which was significantly higher than the basal HCO P_app_ of 0.41–0.44 × 10^−7^ cm/s (*p* = 0.0012). Incubation of 10 ng/mL of TNFα and IFNγ caused a significant increase in the P_app_ of FD4 in both HIO- and HCO-epithelium (Figure 3B). A concentration-dependent response was observed when treated with 1 ng/mL, an increase (*p* < 0.05) was observed in all lines in HIO- and HCO-epithelium with the exception of 03i HIO (*p* = 0.08). This enhanced permeability was not due to cytokine induced cytotoxicity as assessed by the MTS cell viability assay (Figure 3C). This data again demonstrated that HCO-epithelium had an intrinsically increased TEER compared to HIO-epithelium, was physiologically responsive to proinflammatory cytokines in a concentration-dependent manner, and confirmed the applicability of this cellular source for permeability studies.

### 2.4. Tight and Adherens Junction Proteins are Downregulated and Mislocalized in HIO- and HCO-Epithelium Treated with Pro-Inflammatory Cytokines

**Figure 4:**
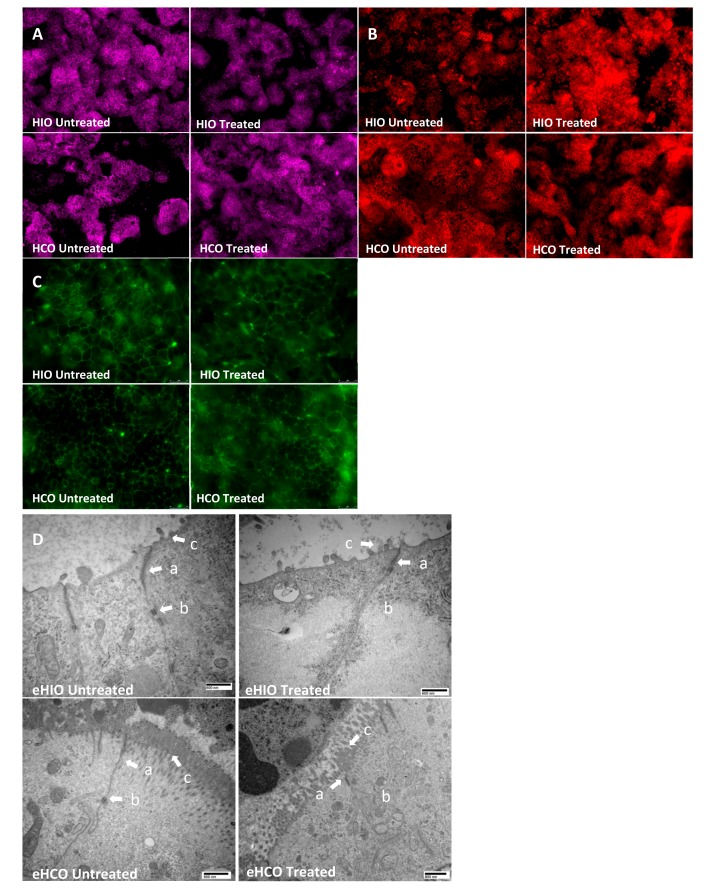
Representative immunofluorescence images of (**A**) JAM-A, (**B**) E-cadherin, and (**C**) ZO-1 in untreated or treated (10 ng/mL TNFα and IFNγ) in HIO- and HCO-derived epithelium grown on Transwell inserts (A and B; 20× and C; 40× magnification. (**D**) Representative transmission electron microscopy images indicate TJ (a) and AJ (b) bundles and microvilli (c) untreated or treated (10 ng/mL TNFα and IFNγ) in eHIO and eHCO.

To further study the effect of TNFα and IFNγ on paracellular permeability, monolayers derived from HIOs and HCOs along with eHIOs and eHCOs, were exposed to these inflammatory cytokines and immunofluorescence along with TEM imaging was carried out. In untreated Transwell cultures containing cells from HIOs and HCOs, there was appropriate localization of JAM-A, ZO-1, and E-cadherin at the bicellular junction (Figure 4). Localization of JAM-A was mostly unaffected by the addition of cytokines in HCO (Figure 4A) while minor internalization was noted in HIO treated epithelium. E-cadherin was significantly internalized and mislocalized in treated HIO- and to a lesser extent in HCO whereby there was minor mislocalization and increased intracellular granularity (Figure 4B). ZO-1 appeared bundled at tricellular junctions and became less continuous and more punctuated in both types of epithelium under treated conditions (Figure 4C). TEM imaging indicated morphological difference between eHIO and eHCO (Figure 4D). Under control conditions, eHIO morphology indicated the presence of sparse microvilli (indicated with c) with apparent tight junction (TJ) protein bundles (indicated with a) and adherens junction (AJ) protein bundles (indicated with b). eHCO also had TJ and AJ protein bundles but had more developed and continuous microvilli on the apical membrane (indicated with a). There was a slight dilation of the paracellular space after the end of the TJ protein bundles in both tissues as expected. Under treated conditions both tissues were noted to have a consistently reduced presence of AJ protein bundles and paracellular edema were observed at times.

**Figure 5:**
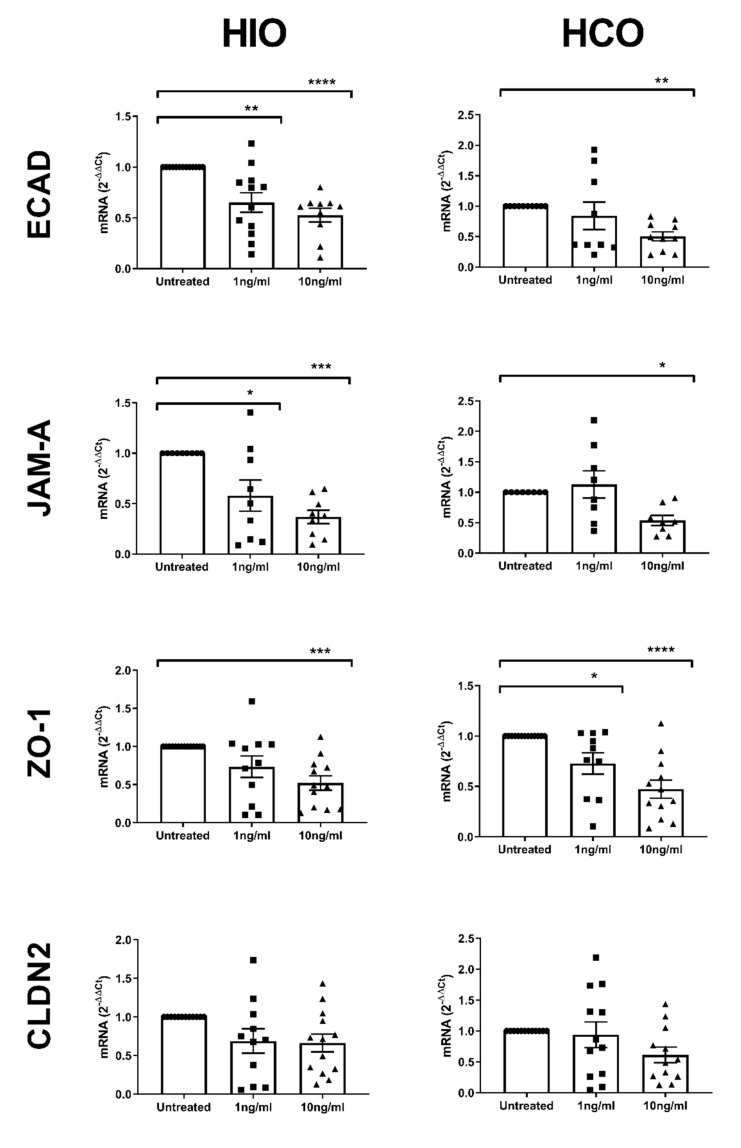
Effect of TNFα and IFNγ (1 and 10 ng/mL) for 72 h on the gene expression of *ECAD*, *JAM-A*, *ZO1* and *CLDN2* as measured by qRT-PCR. Relative gene expression is expressed as the ΔΔ*C*t of GAPDH expression of controls. Two-way ANOVA with Bonferroni’s multiple comparisons; * *p* < 0.05, ** *p* < 0.01, *** *p* < 0.001, **** *p* < 0.0001 compared with respective control. Each value represents the mean ± SEM, *n* = 3, with 3 independent replicates.

To further elucidate the response of these organoids to TNFα and IFNγ exposure, qRT-PCR was carried out. Treatment at 10 ng/mL for 72 h resulted in a decreased expression of *ECAD*, *JAM-A* and *ZO-1* in both eHIO and eHCO (Figure 5). However, 1 ng/mL resulted in more variable results with only a reduction of *ECAD* and *JAM-A* expression in eHIO (*p* < 0.01 and 0.05 respectively) and *ZO-1* in eHCO (*p* < 0.05). Interestingly, *CLDN2* expression was mostly unchanged by 1 and 10 ng/mL exposure. This data suggested that TNFα and IFNγ caused a mislocalization of tight and adherens junction proteins and reduced mRNA expression, resulting in the observed increase in paracellular permeability.

## 3. Discussion

Increased intestinal permeability in both the small and large intestine is associated with IBD and this may be caused by either genetic or environmental components [1,3]. Our goal was to develop a personalized platform that would allow each of these components to be studied under a tightly controlled milieu. To this end, we generated iPSCs from control individuals and a range of IBD patients and firstly directed them to form HIOs, then seeded them onto Transwell inserts to examine their intrinsic permeability under control conditions and subsequently in response to inflammatory cytokines. Previous reports show that there is increased permeability in IBD patients, even in remission states, which supports the hypothesis that there is an intrinsic altered permeability in these individuals [2,3]. While we observed no difference in permeability between our groups (Figure 1A), our low number of subjects coupled with the enormous heterogeneity observed in IBD prevents us from proving or disproving this hypothesis but the finding that P_app_ of FD4 can be assessed consistently in multiple patient iPSC-derived lines in Transwell inserts confirms the technical feasibility of this approach and now permits an examination of this hypothesis by including a much larger number of subjects within each group. The basal P_app_ of FD4 (0.57–0.92 × 10^−7^ cm/s) compared favorably with human small intestine (0.5–2.1 × 10^−7^ cm/s) recordings in Ussing chambers [33,34]. Additionally, our P_app_ values along with our basal TEER values (404–615 Ω·cm^2^; Figure 2B) also compared favorably to a recent study which reported a basal TEER of 420 Ω·cm^2^ and P_app_ value of 0.12 × 10^−6^ cm/s using iPSC derived intestinal epithelial cell monolayers generated by *CDX2* transduction [35].

Numerous studies have used the cytokines TNFα and IFNγ to examine for increased permeability using adenocarcinoma cell lines and indeed our lab previously reported that IFNγ stimulated HIOs grown on a microfluidic device were physiologically responsive via pSTAT1 activation and had increased permeability when TNFα was co-incubated [36,37,38,39]. In the Transwell model, we observed significant increases in P_app_ values across all lines (1.94–3.81 × 10^6^ cm/s) (Figure 1A) and while there were no significant differences among the groups, an adequately powered study may ultimately reveal differences among them. Nevertheless, this proves that the intrinsic permeability under basal culturing conditions are similar to those reported elsewhere and responses to inflammatory cytokines associated with IBD can be measured in this modelling system.

Two papers previously reported the development of iPSC-derived colonic organoids, which were differentiated by the addition of BMP2 (a TGF-β family member which activates SMAD1/5/8 signaling) [15] or LDN193189 (a BMP pathway inhibitor of ALK1/2/3/6 signaling) [16]. To confirm the generation of colonic tissue from iPSCs, we directed a subset of our iPSCs to form HCOs utilizing the protocol by Munera et al. [15] and assessed for the expression of colonic regional markers along with additional permeability readings. Previous reports have shown there is increased expression of *CD147*, *CA2*, and *CDX2* in the human large intestine as compared to the small intestine and we observed similar findings in our eHCOs (Figure 2A) [16,40,41]. Surprisingly, there were no significant differences in *MCT1* and *SATB2* between the organoid groups which conflicts with previous reports (Figure 2A) [15,42,43]. With regards to *MCT1*, it was reported [43] that there was a significant decrease in expression of *MCT1* in the sigmoid colon as compared to ascending, transverse and descending colon and may suggest that our iPSC-derived colonic tissue represents a more distal segment of the large intestine. Expression levels of *SATB2* were extremely low and may not be reflective of its protein expression within our system. We also assessed for markers that are equally expressed throughout the intestinal tract and found that *GATA6*, *ECAD*, and *LGR5* were similarly expressed in both groups of organoids [44,45]. The finding that the basal P_app_ of 0.41–0.44 × 10^−7^ cm/s of HCOs (Figure 3B) compares favorably to previous reports using Caco-2 monolayers (0.2–0.5 × 10^−7^ cm/s) and human colon (0.1–0.2 × 10^−7^ cm/s) recording in Ussing chambers [27,30,33,46]. The 2–2.5 fold decrease in intestinal permeability in colonic epithelium favorably reflects previously published data that show that there is 2–4 fold decrease in permeability in both rat and human explants of small and large intestinal cells (Figure 2B, Table 1). Finally, TEM images obtained in this study indicated our colonic organoids possess morphologically characteristic microvilli found in colonic tissue, while our intestinal organoids reflect small intestinal crypt cells with sparse microvilli buds (Figure 4D) [47]. Overall, significantly increased expression of colonic genes, decreased intestinal permeability and morphological characteristics observed by TEM confirms the previously published report by Munera et al. [15], suggesting a robust method for colonic differentiation.

To further examine the effect of cytokines in our modelling system, we examined for changes in gene expression or mislocalization of tight/adherens junction proteins. As in previous reports using adenocarcinoma cancer cell lines, we similarly observed mislocalization of ZO1 and E-cadherin in treated Transwells containing epithelial cells derived from either HIOs or HCOs (Figure 4B,C) [37,38]. While there were minor changes in the localization of E-cadherin in the treated eHCO which were relatively similar to a previous report [48], there was internalization of E-cadherin in eHIO suggesting differential response in various intestinal segments. Significant changes in the gene expression of tight and adheren junctions have been reported in response to various inflammatory associated with IBD [49,50] and we saw similar significant reductions in expression in our system (Figure 5). The results demonstrated that changes in tight and adherens junction proteins could be observed in our modelling system.

To conclude, we have confirmed that iPSCs-derived intestinal and colonic organoids can be incorporated into Transwell inserts to study barrier integrity under basal and inflammatory conditions. We showed that HIO- and HCO-epithelium have differences in gene expression, electrophysiology, and morphology. Exposure to pro-inflammatory cytokines induced changes in tight and adherens junction gene expression, and protein localization resulting in increased paracellular permeability of FD4. Therefore, this is an appropriate physiologically responsive model to study barrier dysfunction and the role of the epithelium in IBD and VEO-IBD.

## 4. Materials and Methods

### 4.1. Ethical Statement

All the cell lines and protocols in the present study were carried out in accordance with the guidelines approved by the stem cell research oversight committee (SCRO) and institutional review board (IRB) at the Cedars-Sinai Medical Center under the auspices IRB-SCRO Protocol Pro00027264 (IBD Research using Stem Cells).

### 4.2. Cell Lines and Culturing

Seven iPSC lines, CS03iCTR-n1 (03i; control healthy patient), CS688iCTR-n5 (688i; control healthy patient), CS194iIBDn3EBV (194i; adult IBD patient), CS932iIBDn4EBV (932i; adult IBD patient), CS970iIBDn2EBV (970i; adult IBD patient), CS162iCD-n1 (162i; pediatric VEO-IBD patient) and CS269iCD-n3 (269i; pediatric VEO-IBD patient) were obtained from the iPSC Core at Cedars-Sinai. All lines were fully characterized and were confirmed to be karyotypically normal. All iPSC lines were maintained in an undifferentiated state on Matrigel coated plates in mTeSR1 media (Stem Cell Technologies) under feeder free conditions. iPSC cultures were tested monthly for mycoplasma contamination.

### 4.3. Organoid Differentiation From iPSCs

The generation of HIOs and HCOs from iPSCs involves a multistep technique whereby iPSCs were directed to form definitive endoderm, hindgut structures and ultimately organoids. To induce definitive endoderm formation, iPSCs were cultured with a high dose of Activin A (100 ng/mL, R&D Systems, Minneapolis, MN, USA) with increasing concentrations of FBS over time (0%, 0.2% and 2% on days 1, 2 and 3 respectively) in RPMI 1640 (Gibco). Wnt3A (25 ng/mL, R&D Systems, Minneapolis, MN, USA) was also added on the first day of endoderm differentiation. To induce hindgut formation, cells were cultured in Advanced DMEM/F12 (Gibco, Gaithersburg, MD, USA) with 2% FBS and FGF4 (500 ng/mL, R&D Systems, Minneapolis, MN, USA) and CHIR99021 (3 µM, Tocris, Ellisville, MO, USA). After 4 days, free floating epithelial spheres and loosely attached epithelial tubes became visible and were harvested. These epithelial structures were subsequently suspended in Matrigel and then overlaid in intestinal medium (to generate HIOs) containing CHIR99021 (2 µM, Tocris, Ellisville, MO, USA), noggin and EGF (both 100 ng/mL, all R&D Systems, Minneapolis, MN, USA) and B27 (1×, Invitrogen) or colonic medium (to generate HCOs) containing BMP2 (100 ng/mL, Stem Cell Technologies, Vancouver, BC, Canada), EGF (100 ng/mL) and B27 (1×) both in Advanced DMEM F/12 with penicillin streptomycin and L-glutamine (5% *v*/*v*). Media for the HCOs was changed after 3 days with only EGF and B27 remaining. HIOs and HCOs were passaged after 10 days.

### 4.4. Cell Sorting and Transwell Seeding and Monolayer Maintenance

HIOs and HCOs were removed from Matrigel and washed 3–4 times in PBS and subsequently incubated in TrypLE Express (Life Technologies, Camarillo, CA, USA) for 20 min (HIOs) and 40 min (HCOs) until the organoids were completely disassociated to a single cell suspension. These cells were then passed through a 30 µm filter and stained with CD326 (EpCAM; Biolegend, San Diego, CA, USA) for 30 min at 4 °C. The cells were then sorted using magnetic-activated cell sorting (MACS; Miltenyi Biotec, San Diego, CA, USA) and the EpCAM+ cells were collected and resuspended at a density of 2 × 10^6^ cells/mL in intestinal/colonic media containing ROCK inhibitor (10 µM, Tocris, Ellisville, MO, USA), SB202190 (10 µM, Tocris, Ellisville, MO, USA) and A83-01 (500 nM, Tocris, Ellisville, MO, USA). Cells were seeded at a density of 2 × 10^5^ cells/well on a 0.33 cm^2^ Transwell filter (polycarbonate, pore size 0.4 µm) (Corning, Tewksbury, MA, USA) and grown for 18 days. After 24 h, unadhered cells were removed and replaced with fresh intestinal/colonic media containing SB202190 and A83-01. Transepithelial electrical resistance (TEER, Ω·cm^2^) was measured across the monolayers using an EVOM^®^ voltohmmeter with a chopstick electrode (EVOM^®^, WPI) at days 6, 12 and 18 to confirm barrier integrity.

### 4.5. Transwell Cytokine Challenge: Permeability and Cytotoxicity Assay

At day 18 monolayers were treated with TNFα and IFNγ: 10 ng/mL, 1 ng/mL or untreated for 3 days. TEER was measured each day, and after 3 days transport experiments were carried out to assess barrier dysfunction. Apical-to-basolateral (A to B) transport of FD4 (Sigma-Aldrich, Milwakee, WI, USA) was examined across monolayers. The transport buffer consisted of HBSS supplemented with 12.5 mM glucose and 25 mM HEPES (pH 7.4). Intestinal/colonic media was replaced with HBSS and equilibrated for 30 min in transport buffer. At time zero, FD4 (10 µg/mL) was added to the apical side. Basolateral samples were taken every 30 min for 120 min and apical samples were taken at 0 and 120 min in order to calculate the P_app_. Withdrawn samples were replaced with an equal volume of fresh HBSS. Fluorescence was measured in a spectrofluorometer (EnVision 2104, Perkin Elmer, San Jose, CA, USA) with excitation wavelength of 490 nm and an emission wavelength of 525 nm. Basal TEER values were required to be > 250 Ω.cm^2^ in order to be included. The P_app_ for FD4 was calculated according to the following equation:

(1) $$P_{app}=\frac{dQ}{dt}\;x\;\frac{1}{A\;.\;C_{0}}$$

where dQ/dt is the transport rate across the epithelium, *A* is the surface area (0.33 cm^2^) and C_0_ is the starting concentration of flux marker on the apical side. After the final time point for the permeability experiments, the Transwell was washed with transport buffer and the monolayers were treated with MTS (3-[4,5-dimethylthiazol-2-yl]-5-[3-carboxymethoxyphenyl]-2-[4-sulfophenyl]-2H-tetrazolium) to assess cytotoxicity induced by TNFα and IFNγ and exposure. Transwells were incubated with MTS for 4 h at 37 °C in a humidified incubator with 5% CO_2_ and 95% O_2_. Optical density was measured at 490 nm. Each value presented was normalized against untreated control. Transport experiments were run in triplicate (*n* = 3) with a minimum of 3 independent replicates.

### 4.6. Epithelial only-HIO/HCO Analysis: Regional Markers and Cytokine Challenge

EpCAM+ cells were suspended at a density of 5 × 10^4^ cells/mL in Matrigel and overlaid with intestinal/colonic media containing ROCK inhibitor (10 µM, Tocris, Ellisville, MO, USA), SB202190 (10 µM, Tocris, Ellisville, MO, USA) and A83-01 (500 nM, Tocris, Ellisville, MO, USA) to generate epithelial only- HIO (eHIO) and HCO (eHCO) lacking any mesenchymal cells. After 7–10 days the eHIO/eHCO were treated with TNFα and IFNγ: 10 ng/mL, 1 ng/mL or untreated for 3 days. Treated eHIOs and eHCOs were removed from Matrigel and washed 3–4 times in PBS and lysed with RLT Lysis Buffer and extracted with RNeasy mini kit (Qiagen, Carlsbad, CA, USA). cDNA was generated from 1 µg of RNA using the Omniscript RT Kit (Qiagen, Carlsbad, CA, USA). Quantitative real-time polymerase chain reaction was performed using SYBR Select Master Mix (Applied Biosystems, Carlsbad, CA, USA) on a BioRad CFX384 Real-Time System. Primer sequences as follows (in Table 2):

### 4.7. Image Analysis: Immunofluorescence and Transmission Electron Microscopy

For immunofluorescence analysis eHIOs, eHCOs and Transwell inserts were fixed in 4% paraformaldehyde (Electron Microscopy Sciences, Hatfield, PA, USA) and washed with PBS. Samples were blocked in 10% normal donkey serum (Jackson ImmunoResearch, West Grove, PA, USA) with 0.5% Triton X-100 and incubated with primary antibodies overnight at 4 °C. Primary antibodies were as follows: ZO-1 mouse monoclonal (Thermo Fisher, Carlsbad, CA, USA), JAM-A mouse monoclonal (Santa Cruz Biotechnology, Santa Cruz, CA, USA), and E-cadherin polyclonal (R&D Systems, Minneapolis, MN, USA). Inserts were rinsed and incubated in species specific AF488 or AF594-conjugated secondary antibodies (Life Technologies, Carlsbad, CA, USA) were imaged using a Leica DM6000 B microscope. For transmission electron microscopy (TEM) eHIOs and eHCOs were fixed in 3% glutaraldehyde. Ultrathin sections of plastic-embedded tissue were processed for electron microscopy as previously described [51]. TEM images at indicated magnification were obtained using JEM-100CX transmission electron microscope (JEOL, Tokyo, Japan).

### 4.8. Statistical Analyses

All data are represented as mean ± standard error of the mean (SEM) unless otherwise stated. Statistical analysis was carried out using Prism-7 software (GraphPad) using one-way ANOVA and two-way ANOVA with Dunnett’s and Bonferroni’s post hoc test respectively. A significant difference was considered present if *p* < 0.05.

## Figures and Tables

**Table 1:** Comparison of HIO- and HCO-derived epithelium TEER and published values from human and rat small and large intestinal tissue.

Tissue Source	TEER Values (Ω.cm^2^)	Fold Difference	References
Human small intestine	23–45	-	[20,21,22,23]
Human colon	103–174	4-fold	[23,24,25,26,27]
Rat small intestine	30–37	-	[22,28,29]
Rat colon	84–116	3-fold	[27,30,31,32]
HIO	478 ± 24	-	
HCO	1268 ± 50	3-fold	

**Table 2:** Primer sequences for intestinal regional expression and tight junctions.

Gene	Forward	Reverse
CD147	ACTCCTCACCTGCTCCTTGA	GTCCACCTTGAACTCCGTTTTC
CDX2	TTCCTCTCCTTTGCTCTGCG	AGTCGCTACATCACCATCCG
CA2	AAACACAACGGACCTGAGCA	GGAAACAGACAGGGGCTTCA
SATB2	GCAAAGCCAAGCACCAGAAA	TAAAACGCACAGGGACTGCT
GATA6	TGCTATTACCAGAGCAAGTCTTTG	TGTGCAATGCTTGTGGACTC
MCT1	CCACCACTTTTAGGTCGGCT	TGCCCATGCCAATGAAGAGA
LGR5	GAACACTGACTCTGAATGGTGC	ACGGTTTGAGGAAGAGATGAGA
ZO-1	ACAGCAATGGAGGAAACAGC	CCCCACTCTGAAAATGAGGA
JAM-A	TCATATTGGCGATCCTGTTG	AGGCACAGGACAACTTCACA
ECAD	GCCGAGAGCTACACGTTCAC	GTCGAGGGAAAAATAGGCTG
